# Predictive Modeling of Morbidity and Mortality in Patients Hospitalized With COVID-19 and its Clinical Implications: Algorithm Development and Interpretation

**DOI:** 10.2196/29514

**Published:** 2021-07-09

**Authors:** Joshua M Wang, Wenke Liu, Xiaoshan Chen, Michael P McRae, John T McDevitt, David Fenyö

**Affiliations:** 1 Institute for Systems Genetics NYU Grossman School of Medicine New York, NY United States; 2 Department of Biochemistry and Molecular Pharmacology NYU Grossman School of Medicine New York, NY United States; 3 Vilcek Institute of Graduate Biomedical Sciences NYU Grossman School of Medicine New York, NY United States; 4 Department of Medicine NYU Grossman School of Medicine New York, NY United States; 5 Department of Biomaterials Bioengineering Institute New York University New York, NY United States; 6 NYU Langone Health New York, NY United States

**Keywords:** COVID-19, coronavirus, SARS-CoV-2, predictive modeling, New York City, prediction, model, machine learning, morbidity, mortality, hospital, marker, severity, symptom, decision making, outcome

## Abstract

**Background:**

The COVID-19 pandemic began in early 2021 and placed significant strains on health care systems worldwide. There remains a compelling need to analyze factors that are predictive for patients at elevated risk of morbidity and mortality.

**Objective:**

The goal of this retrospective study of patients who tested positive with COVID-19 and were treated at NYU (New York University) Langone Health was to identify clinical markers predictive of disease severity in order to assist in clinical decision triage and to provide additional biological insights into disease progression.

**Methods:**

The clinical activity of 3740 patients at NYU Langone Hospital was obtained between January and August 2020; patient data were deidentified. Models were trained on clinical data during different parts of their hospital stay to predict three clinical outcomes: deceased, ventilated, or admitted to the intensive care unit (ICU).

**Results:**

The XGBoost (eXtreme Gradient Boosting) model that was trained on clinical data from the final 24 hours excelled at predicting mortality (area under the curve [AUC]=0.92; specificity=86%; and sensitivity=85%). Respiration rate was the most important feature, followed by SpO_2_ (peripheral oxygen saturation) and being aged 75 years and over. Performance of this model to predict the deceased outcome extended 5 days prior, with AUC=0.81, specificity=70%, and sensitivity=75%. When only using clinical data from the first 24 hours, AUCs of 0.79, 0.80, and 0.77 were obtained for deceased, ventilated, or ICU-admitted outcomes, respectively. Although respiration rate and SpO_2_ levels offered the highest feature importance, other canonical markers, including diabetic history, age, and temperature, offered minimal gain. When lab values were incorporated, prediction of mortality benefited the most from blood urea nitrogen and lactate dehydrogenase (LDH). Features that were predictive of morbidity included LDH, calcium, glucose, and C-reactive protein.

**Conclusions:**

Together, this work summarizes efforts to systematically examine the importance of a wide range of features across different endpoint outcomes and at different hospitalization time points.

## Introduction

The first cluster of SARS-CoV-2 cases was reported in Wuhan, Hubei Province, China, on December 31, 2019. With symptoms remarkably similar to pneumonia, the disease quickly traveled around the world, earning its pandemic status by the World Health Organization on March 11, 2020. Although the first wave has since passed for the hardest-hit regions, such as New York City and most of Asia, a resurgence of cases has already been reported in Europe and a record number of new cases has been tallied in the Midwest and rural United States. As of November 12, 2020, the United States alone logged its highest tally to date, with a 317% growth over the preceding 30 days [1]. COVID-19 is far from seeing the end of its days and there remains a compelling need to prioritize care and resources for patients at elevated risk of morbidity and mortality.

Previous work building machine learning models used patient data from Tongji Hospital in Wuhan, China [2,3]; Zhongnan Hospital in Wuhan, China [4]; Mount Sinai Hospital in New York City, United States [5]; and NYU (New York University) Family Health Center in New York City, United States [6]. Surprisingly, clinical features that were selected varied widely across studies. For example, while McRae et al’s two-tiered model [6] that was trained on 701 patients in New York City to predict mortality was based on actual age, C-reactive protein (CRP), procalcitonin, and D-dimer, Yan et al’s model [2] that was trained on 485 patients from Wuhan selected lactate dehydrogenase (LDH), lymphocyte count, and CRP as the most predictive for mortality. Variations in selected features differed greatly, even when trained to predict similar outcomes on data from patients of the same city. Yao et al’s model [3] was trained on 137 patients from Wuhan, and the final model relied on 28 biomarkers to predict morbidity. Given the differences among prior models, some of which were driven by domain-specific knowledge, we decided to systematically examine the importance of a wide range of features across different endpoint outcomes and at different hospitalization time points.

This study analyzed retrospective polymerase chain reaction (PCR)–confirmed data from inpatients with COVID-19 that were collected at NYU Langone Hospital, spanning from January 1 to August 7, 2020, to predict three sets of clinical outcomes: alive versus deceased, ventilated versus not ventilated, or intensive care unit (ICU) admitted versus not ICU admitted. The clinical information of 3740 patient encounters included demographic data (ie, age, sex, insurance, past diagnosis of diabetes, and presence of cardiovascular comorbidities), vital signs (ie, SpO_2_ [peripheral oxygen saturation], pulse, respiration rate, temperature, systolic blood pressure, and diastolic blood pressure), and the 50 most frequently ordered lab tests in our data set. Models were developed using two methods: logistic regression with feature selection using the least absolute shrinkage and selection operator (LASSO) [7] and gradient tree boosting with XGBoost (eXtreme Gradient Boosting) [8]. An explainable algorithm, such as logistic regression, provides easy-to-interpret insights into the features of importance. Conversely, the larger model capacity of XGBoost better handles data complexities to explore the extent to which predictive performance can be optimized. Together, this study aimed to provide a holistic survey of the clinical underpinnings of disease etiology for patients with COVID-19 admitted to NYU Langone Hospital. In addition, we sought to explore the prospects of building models that are sufficiently competent to be effective decision support tools.

## Methods

### Ethics Statement

An ethics exemption and a waiver were confirmed through the Institutional Review Board (IRB) at NYU Grossman School of Medicine. An IRB self-certification form was completed to ensure that the subsequent research did not fall under human subject research; therefore, no IRB approval was required. The deidentified COVID-19 NYU Langone Database was stripped of all unique identifiers prior to receiving data. In addition, all dates were shifted by an arbitrary number of days for each patient. These safeguards ensured that patient data could not be reidentified; thus, they were not subject to Health Insurance Portability and Accountability Act (HIPAA) restrictions on research use and did not require IRB approval.

### Data Collection

The clinical activity of patients at NYU Langone Hospital was obtained from Epic—electronic medical record system—between January 1 and August 7, 2020. The data were stripped of all unique identifiers (medical record numbers, names, etc) and actual dates were shifted by an arbitrary number of days for each patient, which ensured that no data were subject to HIPAA restrictions and, thus, did not require IRB approval.

### Clinical Data Preprocessing and Cleaning

#### Overview

Our data set contained 206,677 patients who were tested for COVID-19, of which 12,473 (6.0%) tested positive (Multimedia Appendix 1). Not all patients who tested positive sought hospital care, and without vital signs or lab values, these patients were excluded from analysis. In addition, a majority of the 175,507 patients diagnosed with COVID-19 did not receive in-house PCR tests, which makes it difficult to distinguish which hospital encounters were related to seeking COVID-19 treatment. Thus, only patients for which we could confirm a positive PCR test as reported by NYU Langone Hospital were included. The time stamp of the first encounter in which a PCR test returned a positive result was used as the starting date for each patient, and the ending date was determined by either the time of discharge for that encounter or the time of death. The clinical features that were collected for each patient, along with their definitions and additional processing steps, are described in the following subsections.

#### Categorical Features

The categorical features collected for each patient are listed in Textbox 1.

**Categorical features.**

Binned ages—to comply with Health Insurance Portability and Accountability Act restrictions on research use, exact patient ages were removed and binned into predefined ranges, as determined by the Data Handling Committee:

- 0-17 years
- 18-44 years
- 45-64 years
- 65-74 years
- 75+ years

Gender:

- 0 for female
- 1 for male

Insurance type:

- 0 for preferred provider organization
- 1 for exclusive provider organization, health maintenance organization, point-of-service-plan, indemnity, Medicare, Medicare managed care, no fault, and workers’ compensation
- 2 for Medicaid and Medicaid managed care

Diabetes:

- 1 for any past diagnosis mentioning diabetes
- 0 otherwise

Cardiovascular comorbidities:

- 1 for any of the following ICD-10 (International Statistical Classification of Diseases and Related Health Problems, 10th Revision) diagnosis codes: I10-I16 (hypertensive diseases), I20-I25 (ischemic heart diseases), I50 (heart failure), I60-I69 (cerebrovascular diseases), and I72 (other aneurysms)
- 0 otherwise

#### Continuous Features

For each of the following continuous features (Textbox 2), multiple periodic measurements were recorded for each patient by vital signs monitors. Measurements were binned into 24-hour windows that began from time of hospitalization. Within each window, values were averaged. Values were then standardized to a mean of 0 and variance of 1. For each day, encounters without all features listed in Textbox 2 were removed and were not imputed.

**Continuous features.**

- SpO_2_ (peripheral oxygen saturation) (%)
- Pulse (bpm [beats per minute])
- Respiration rate (bpm)
- Temperature (°F)
- Systolic blood pressure (mm Hg)
- Diastolic blood pressure (mm Hg)

#### Outcomes

The outcomes for each patient are listed in Textbox 3.

**Patient outcomes.**

Living status:

- 0 for alive
- 1 for dead

Ventilation at any point during hospitalization:

- 0 for no (did not receive any form of ventilation or only received noninvasive treatments; eg, nasal cannula, nonrebreather mask, etc)
- 1 for yes (received mechanical ventilation treatment)

Intensive care unit admission for any duration during hospitalization—criteria determined by medical triage team, balanced between disease severity and hospital resource availability:

- 0 for no
- 1 for yes

### Lab Data Selection and Cleaning

Lab tests with at least 50% completeness during the first 24 hours for all encounters were considered. Of the 54 lab tests meeting these requirements, the estimated glomerular filtration rate—non-African and African American—was removed due to the formula’s dependency on lab features already selected (ie, creatinine). In addition, the placeholders for ordering a complete blood count with differential test and a COVID-19 PCR test were also removed. Missing lab values were imputed using the multivariate imputation by chained equations algorithm. Five imputations were generated using predictive mean matching. After imputation, lab values were shifted up by 1 and log-transformed. Model-building approaches that incorporated lab features had individual models built for each imputation.

### Feature Selection and Model Building

All models were trained with a training data to validation data ratio split of 90:10. Features for logistic regression were selected using LASSO and optimized for a penalty parameter that was 1 standard error above the minimum deviance for additional shrinkage. The XGBoost parameters were identified using a hyper-parameter search within the following constraints: nrounds=1000; η=0.3, 0.1, or 0.01; max_depth=2, 3, 4, 5, 6, 7, or 8; min_child_weight=0 to 1, by 0.1 increments; and γ=0 to 1, by 0.1 increments. To account for class imbalance, sample-weighted loss was employed when calculating the loss.

For models that were trained on the final day of discharge or death, the performance on predicting outcomes in all preceding days was evaluated on the entire data set rather than just a 10% subset. Data from previous days were not used in the training of these endpoint models and, thus, can all serve as validation data.

### Time Series Modeling

In each feature setting, all variables were combined and missing values at each time point were imputed with the immediate previous value (ie, forward filling). After imputation, time points with incomplete feature measurements were discarded, and each patient record was segmented into nonoverlapping sequences of length 8. Patients were randomly assigned to training, validation, and testing groups in an 8:1:1 ratio for three independent splits. All models were implemented in Python 3.6 (Python Software Foundation) with built-in units in TensorFlow 2 and Keras [9]. Logistic regression was fit as a neural network with the sigmoid output node immediately after the input layer. For multilayer perceptron (MLP), recurrent neural network (RNN), gated recurrent unit (GRU), and long short-term memory (LSTM) models, a hidden layer of size 8 was added, and the time series models (ie, RNN, GRU, and LSTM) were unrolled over eight time points and trained with true labels provided at each step. Five randomly initialized models were trained for all architectures on each training, validation, and testing split. Model performance was evaluated based on all single time point predictions and reported as a mean value across all splits.

### Data Availability

The data that support the findings of this study were obtained from the Medical Center Information Technology (MCIT) at NYU Langone Health, but restrictions apply to the availability of these data and, therefore, they are not publicly available due to specific institutional requirements.

## Results

**Figure 1:**
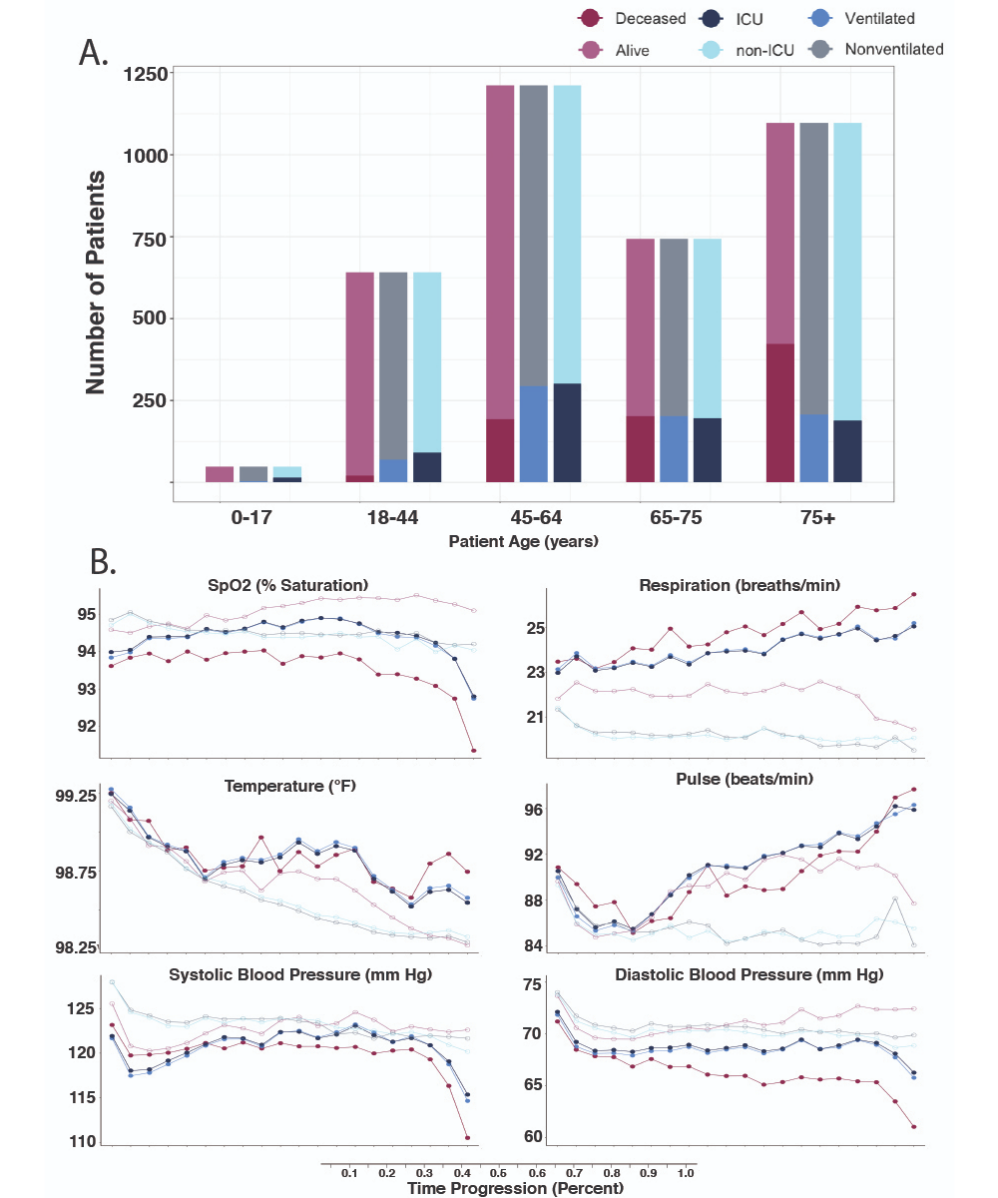
Overview of the clinical data set. A. Patient ages were binned by predefined ranges and the ratio of outcomes compared across age groups. B. For each patient, hospitalization stay was normalized by length of stay and segmented into 5% windows. Within each window, all values for the measured clinical variable were averaged. Each line is colored by the six possible outcomes. ICU: intensive care unit; SpO_2_: peripheral oxygen saturation.

More than half of all patients in our data set were over the age of 65 years, with pediatric patients (0-17 years) having the lowest representation (Figure 1A and Multimedia Appendix 2). Generally, the proportion of deceased patients increased with age, peaking at 38.5% (422/1097) for those 75 years and over, 15.9% (193/1211) for those 45 to 64 years, and 0% for pediatric patients. Most patients who were either ventilated or admitted to the ICU belonged to the 65-to-74-years age group, followed by those 45 to 64 years and 75 years and over.

Aggregation of values for commonly acquired clinical metrics over normalized time courses offered meaningful insights into disease progression. Each patient’s hospitalization stay was segmented into 5% windows, and clinical metrics were averaged within each bin (Figure 1B). We first examined the difference of average vital sign measurements between cohorts with different outcomes. The value of SpO_2_ was statistically different for all three outcome comparisons in the first 5% of hospitalization time (Wilcoxon test: *P*<.001, *P*<.001, and *P*<.001). Over the clinical time course, the difference in SpO_2_ averages increased the most for those who would become deceased, followed by those who were ICU admitted and ventilated. Differences in respiration rate followed a similar adverse trend, with breaths per minute increasing the most for those who would become deceased, followed by those who were ventilated and ICU admitted. The divergence was present even after accounting for overlapping deceased patients. When considering the subset of patients who survived, ventilated patients had 2.91 more breaths per minute (Wilcoxon test: *P*<.001), and ICU-admitted patients had 2.90 more breaths per minute (Wilcoxon test: *P*<.001). At the beginning of the time course, differences in temperature were small (0.05 °F, 0.11 °F, and 0.06 °F, respectively) and not statistically significant for those who would become deceased (Wilcoxon test: *P*=.13), but differences were statistically significant for those who were ventilated (Wilcoxon test: *P*<.001) or were admitted to ICU (Wilcoxon test: *P*<.001). Pulse differences at the beginning were not significantly different for those who were ventilated (Wilcoxon test: *P*=.29) but were significantly different for those who would become deceased (Wilcoxon test: *P*<.001) and ICU admitted (Wilcoxon test: *P*<.001). Systolic and diastolic blood pressure values were continuously lower for patients with worse outcomes in this data set.

To assess the effectiveness of these vital signs to triage clinical outcomes, only data collected in the first 24 hours after admission were initially considered. Specifically for the ventilation outcome, respiration rates and SpO_2_ levels may be influenced by the time point when mechanical ventilation was administered. Of 3740 encounters, 7.0% (262/3740) were ventilated within the first 24 hours of admission. To assess the bias that early administration of mechanical ventilation during the first 24 hours may have on respiration rate and SpO_2_ levels, the distribution of values was compared against a filtered subset containing only values recorded prior to the start of ventilation. At the per-encounter level, the difference in respiration rates (Wilcoxon test: *P*=.26; Multimedia Appendix 3, plot A) and SpO_2_ levels (Wilcoxon test: *P*=.20; Multimedia Appendix 3, plot B) were not significantly different. Given (1) that 93.0% (3478/3740) of encounters were not influenced by early ventilation treatment, (2) the insignificant difference in distributions, and (3) the desire to keep feature selection consistent across models, all values recorded within the first 24 hours were included. For each encounter, continuous features with multiple recordings (ie, SpO_2_, pulse, respiration rate, temperature, systolic blood pressure, and diastolic blood pressure) were averaged and then standardized to a mean of 0 and a standard deviation of 1.

**Figure 2:**
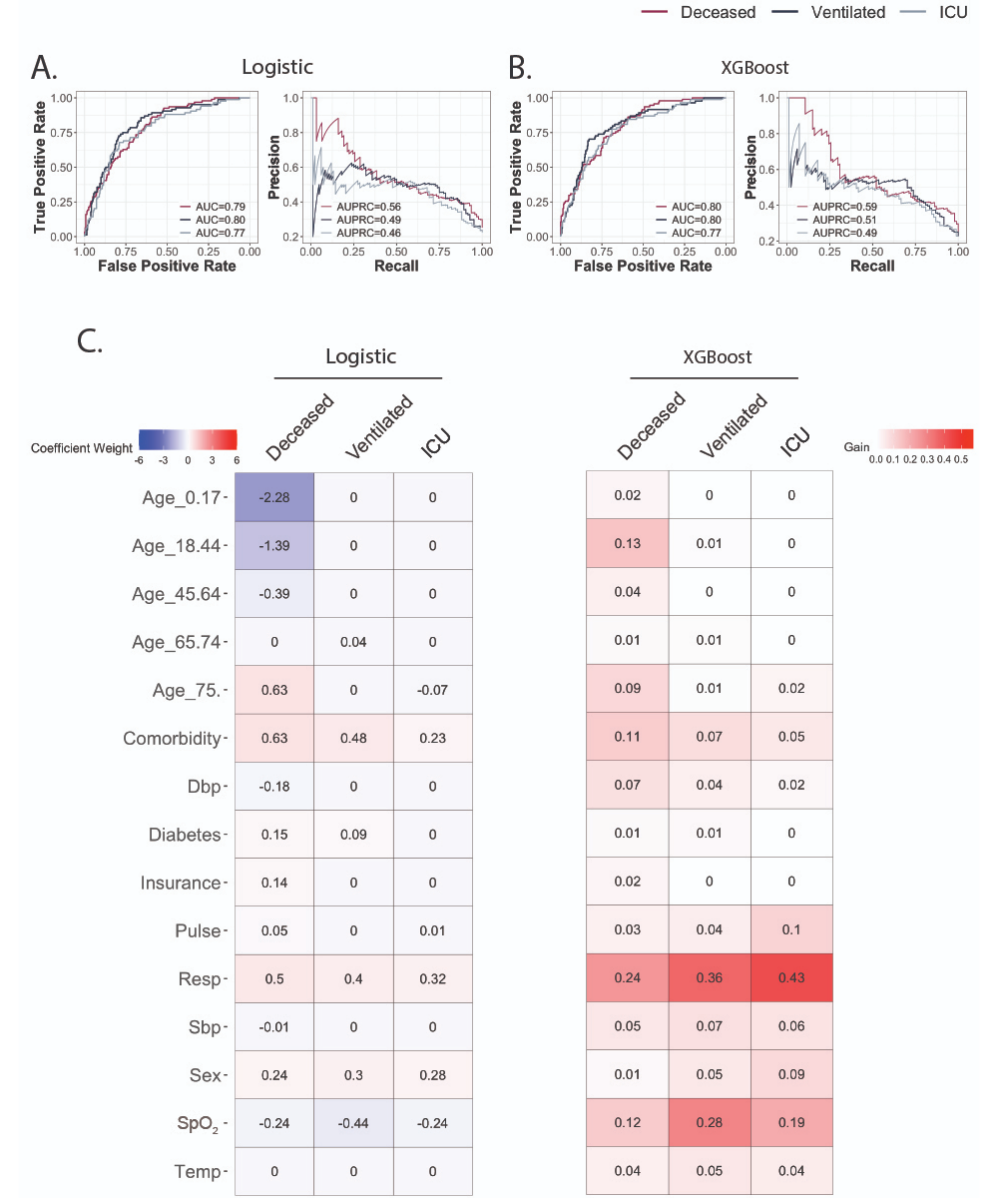
Predictive performance using clinical data from the first 24 hours. A. Receiver operating characteristic (ROC) curve and precision-recall curve (PRC) for logistic regression model. B. ROC curve and PRC for XGBoost (eXtreme Gradient Boosting) model. C. Coefficient weights for the logistic model are recorded on the left. Model performance gains for XGBoost are listed on the right. AUC: area under the curve; AUPRC: area under the precision-recall curve; Dbp: diastolic blood pressure; ICU: intensive care unit; Resp: respiration rate; Sbp: systolic blood pressure; SpO_2_: peripheral oxygen saturation; Temp: temperature.

For logistic regression, features were selected using LASSO with 10-fold cross-validation. Grid search was used to optimize XGBoost parameters (Multimedia Appendix 4). When trained on data from the first 24 hours, the logistic model had area under the curve (AUC) performances of 0.79, 0.80, and 0.77; specificities of 59%, 78%, and 79%; and sensitivities of 86%, 74%, and 68%, respectively (Figure 2A). XGBoost performed similarly, with AUC performances of 0.80, 0.80, and 0.77; specificities of 59%, 83%, and 69%; and sensitivities of 86%, 70%, and 77%, respectively (Figure 2B).

In both logistic regression and gradient tree boosting settings, features of importance varied across clinical outcomes (Figure 2C). For logistic regression models of the three outcomes, respiration rate, SpO_2_, and cardiovascular comorbidity were among predictive features, but age groups were selected only for predicting mortality. For boosting tree models, feature importance measures showed that respiration rate was consistently the most important feature for all three outcomes, and the age category 18 to 44 years was the second most important feature only for vital status. Respiration rate and SpO_2_ were important for predicting all three outcomes. Differences in temperature were not strongly predictive in any cohort in either model; this finding and temperature’s insignificant difference in the deceased outcome group together suggest that its role in screening for increased disease severity may not be dependable.

**Figure 3:**
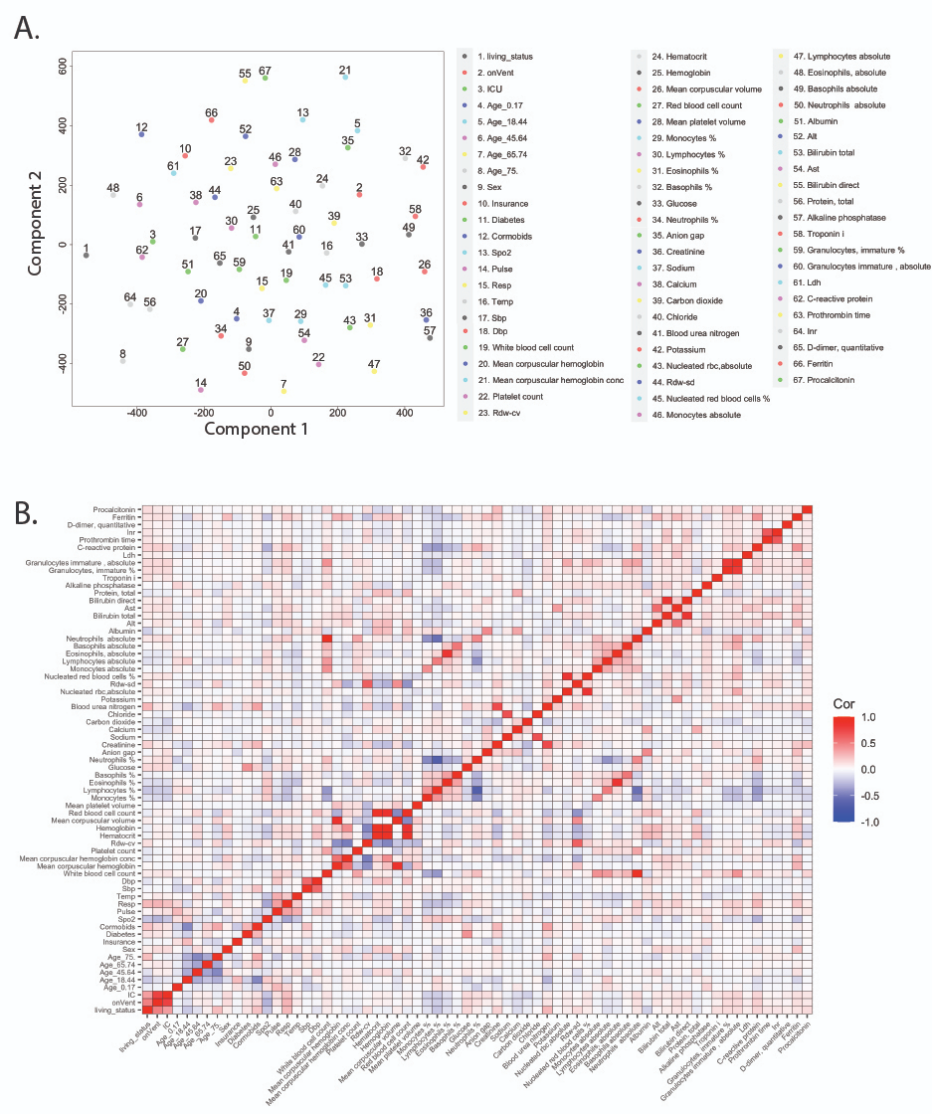
Overview of lab features collected in the first 24 hours. A. A t-distributed stochastic neighbor embedding plot based on previously collected clinical features and new lab values. B. Pairwise Pearson correlation heat map. Alt: alanine aminotransferase; Ast: aspartate aminotransferase; Dbp: diastolic blood pressure; ICU: intensive care unit; Inr: international normalized ratio; Ldh: lactate dehydrogenase; rbc: red blood cell; Resp: respiration rate; Rdw-cv: red cell distribution width–coefficient of variation; Rdw-sd: red cell distribution width–standard deviation; Resp: respiration rate; Sbp: systolic blood pressure; SpO_2_: peripheral oxygen saturation; Temp: temperature.

The 50 most frequently collected lab values and their relative importance were also studied. A t-distributed stochastic neighbor embedding plot (Figure 3A) suggests lack of clustering among lab features and overall low correlation (Figure 3B) in pairwise comparisons (|μ|=0.08; |σ|=0.10). Local pockets of correlation (|*r*|≥0.83) were identified between hemoglobin, hematocrit, and red blood cell count; absolute neutrophils and white blood cell count; and bilirubin direct and bilirubin total. Each of these sets measures variables that are clinically interdependent and, thus, expected.

**Figure 4:**
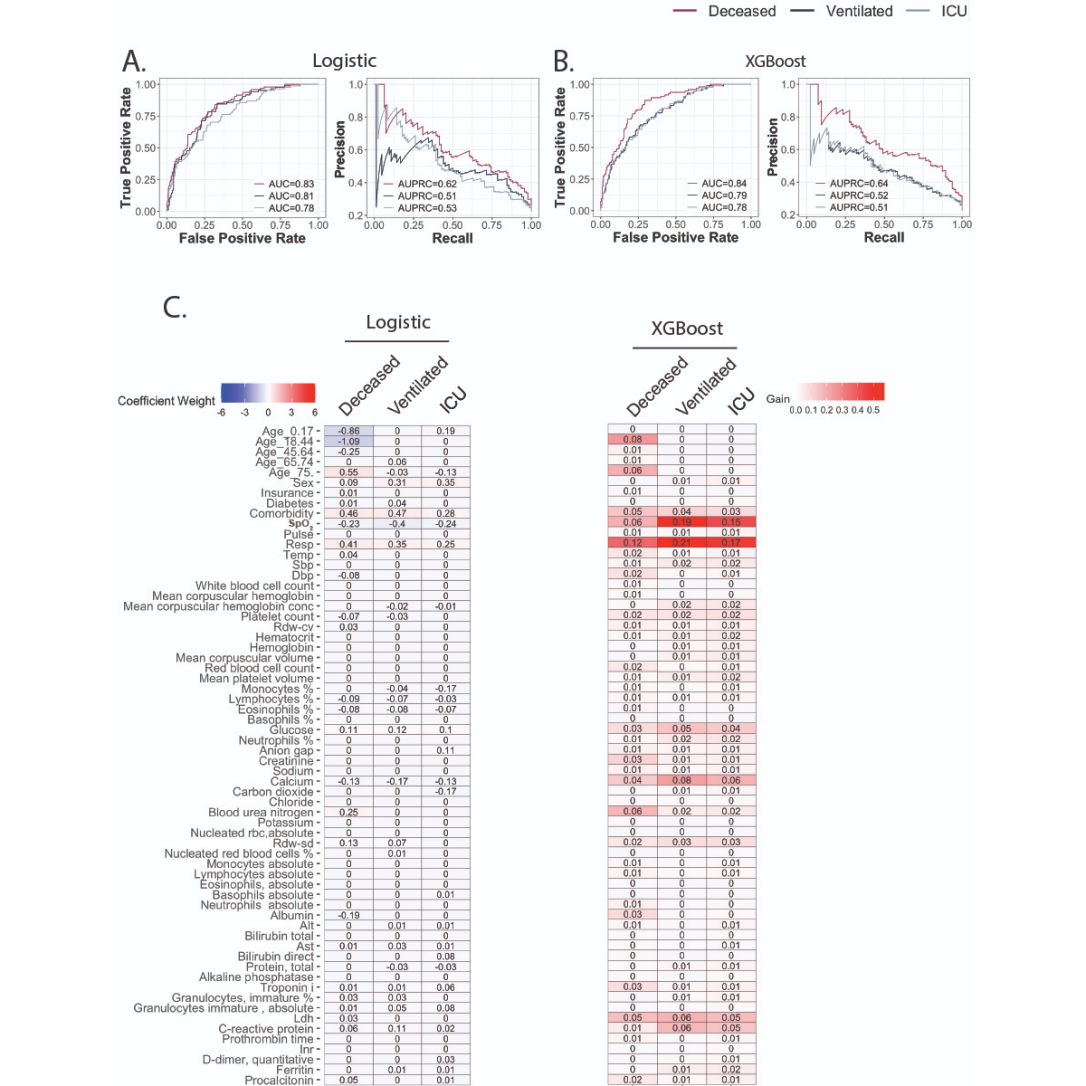
Predictive performance after incorporating lab features. A. Receiver operating characteristic (ROC) curve and precision-recall curve (PRC) for logistic regression model. B. ROC curve and PRC for the XGBoost (eXtreme Gradient Boosting) model. C. Coefficient weights for the logistic model are recorded on the left. Model performance gains for XGBoost are listed on the right. Alt: alanine aminotransferase; Ast: aspartate aminotransferase; AUC: area under the curve; AUPRC: area under the precision-recall curve; Dbp: diastolic blood pressure; ICU: intensive care unit; Inr: international normalized ratio; Ldh: lactate dehydrogenase; rbc: red blood cell; Resp: respiration rate; Rdw-cv: red cell distribution width–coefficient of variation; Rdw-sd: Red cell distribution width–standard deviation; Sbp: systolic blood pressure; SpO_2_: peripheral oxygen saturation; Temp: temperature.

Incorporating lab features into the predictive models marginally improved performance. Logistic regression had AUC performances of 0.83, 0.81, and 0.78; specificities of 68%, 70%, and 69%; and sensitivities of 85%, 83%, and 74%, respectively (Figure 4A). The XGBoost model performed better, with AUC increasing to 0.84, 0.79, and 0.78; specificities of 71%, 72%, and 65%; and sensitivities of 83%, 73%, and 78%, respectively (Figure 4B). For logistic regression, blood urea nitrogen (BUN) and albumin were among the lab features (Figure 4C) that were predictive of mortality. The XGBoost model found the most performance gain from BUN and LDH. Feature importance for predicting ventilation or ICU admission differed between models. For ventilation, logistic regression selected calcium, glucose, and CRP with large absolute coefficient values, while XGBoost identified calcium, glucose, CRP, and LDH as important features. For those admitted to ICU, XGBoost benefited from the same lab features, while monocyte percentage and carbon dioxide were additionally selected for by logistic regression. Of note, for XGBoost, no lab feature showed a higher importance measure than did respiration rate and SpO_2_ for all three outcomes.

**Figure 5:**
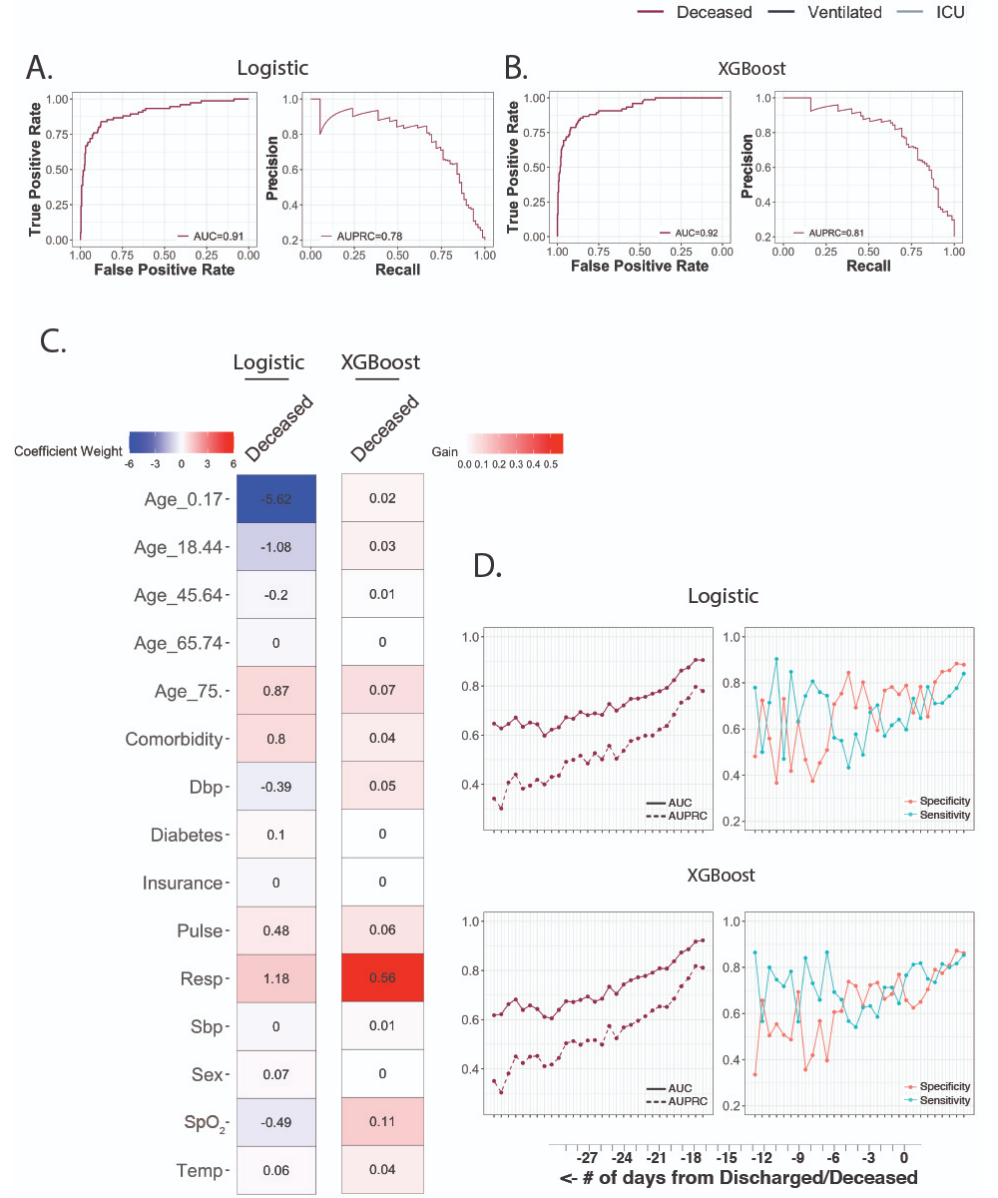
Predictive performance of deceased using clinical data from the final 24 hours. A. Receiver operating characteristic (ROC) curve and precision-recall curve (PRC) for the logistic regression model. B. ROC curve and PRC for the XGBoost (eXtreme Gradient Boosting) model. C. Coefficient weights for the logistic model are recorded on the left. Model performance gains for XGBoost are listed on the right. D. Performance of models to predict deceased outcome was assessed using clinical data from the preceding 30 days. Plots track the area under the curve (AUC), area under the precision-recall curve (AUPRC), specificity, and sensitivity when using the threshold that maximized the sum of the sensitivity and specificity (Youden’s J statistic). Dbp: diastolic blood pressure; ICU: intensive care unit; Resp: respiration rate; Sbp: systolic blood pressure; SpO_2_: peripheral oxygen saturation; Temp: temperature.

Finally, models trained on data collected in the last 24 hours excelled at predicting which patients would become deceased. The logistic regression model (Figure 5A) had an AUC performance of 0.91, specificity of 88%, and sensitivity of 84%. The XGBoost model (Figure 5B) had an AUC performance of 0.92, specificity of 86%, and sensitivity of 85%. The importance of respiration rate increased for XGBoost (Figure 5C), accounting for more than 50% of the gain. Values of SpO_2_ and being aged 75 years and over were the next most important features.

Using the same coefficients and tree weights and structures, both models were assessed based on clinical data from the preceding 30 days (Figure 5D). With cutoffs of 0.80 for AUC, and 70% for specificity and sensitivity, logistic regression was able to predict a deceased outcome 4 days in advance (AUC=0.82; specificity=85%; and sensitivity=71%) and 5 days in advance (AUC=0.81; specificity=70%; and sensitivity=75%) for XGBoost. Models were not trained on those ventilated or ICU admitted, as these events are unlikely to occur in the final 24 hours preceding discharge and death. Lab values were not incorporated because few blood tests were ordered in the final 24 hours.

**Figure 6:**
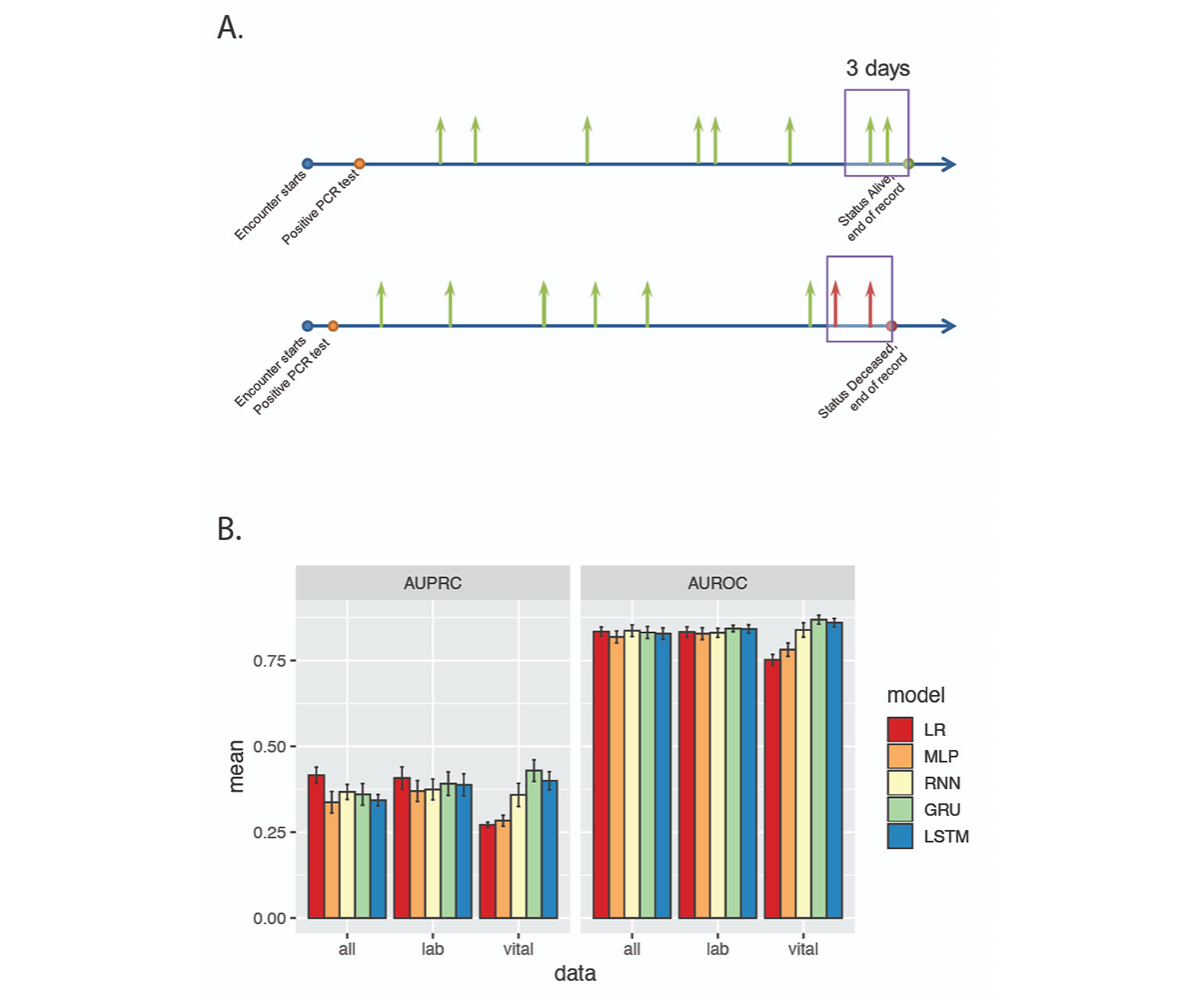
Time series model performance. A. Time series labels for mortality risk. Medical measurements are obtained at time points across the admission period with uneven intervals. Green and red arrows represent time points with negative and positive (0/1) labels. B. Mean values of the area under the precision-recall curve (AUPRC) and area under the receiver operating characteristics curve (AUROC) for five model architectures across three feature settings. GRU: gated recurrent unit; LR: logistic regression; LSTM: long short-term memory; MLP: multilayer perceptron; PCR: polymerase chain reaction; RNN: recurrent neural network.

To explore whether patient status can be dynamically predicted based on past medical data, we also built time series models using simple RNN, GRU, and LSTM architectures and compared the performance metrics to single time point models of logistic regression and MLP. A major goal is to explore whether more complex modeling approaches are able to make accurate and transferrable predictions. The vital status of each patient was converted to a time series that was marked as positive if the time point was within 3 days of the patient becoming deceased (Figure 6A). The time series models (ie, RNN, GRU, and LSTM) were trained to take medical data from eight time points as input and infer the vital status at each point, giving rise to a real-time risk prediction based on historical records. The single time point models (ie, logistic regression and MLP), on the other hand, only used medical data at the current point to make the prediction. Model comparison was carried out with three different feature sets: vital signs only (ie, body temperature, pulse, respiration rate, systolic blood pressure, diastolic blood pressure, and SpO_2_), vital signs and 46 lab results with nonzero coefficients in the single time point LASSO regression model, and vital signs and lab results plus *static* demographic information (ie, sex, age group, diabetic history, and comorbidities) (Multimedia Appendix 5). As the time series data were recorded at uneven and irregular intervals, the progression time (in days) was included in all models as an additional feature. For models only including vital sign features, time series models showed better performance (Figure 6B) compared to single time point models, but performance was comparable among all models when lab results and demographic information was added to the feature set.

## Discussion

Retrospective analysis of patients who tested positive for COVID-19 identified recognizable clinical markers, such as respiration rate and SpO_2_, but also provided insights distinguishing morbidity (ie, ICU-admitted or ventilated outcomes) from mortality (ie, deceased outcome). Our study confirmed canonical risk factors that were previously established (eg, age, respiration rate, and SpO_2_) as predictive of mortality and morbidity, but also uncovered the surprising finding that temperature was not predictive for mortality. In addition, lab markers of physiological stress, including LDH, BUN, and CRP, were found to be important for model prediction, but other canonical indicators, such as procalcitonin, were not.

Our results aligned with previous work [10] analyzing patient data from NYU Langone Health to predict absence of adverse events within a 96-hour window as opposed to negative outcomes. Several features of importance overlapped both studies, notably respiration rate, SpO_2_, LDH, BUN, and CRP. However, other selected features, such as temperature, platelet count, pulse, and eosinophil percentage, were not found to be important in our model.

Although the goal of stratifying patients by disease severity aligned, the different approaches likely explain the differences in variable explanation. Our study differs in that our models were trained only on clinical data from the first 24 hours after admission, as compared to continuously updating predictions when new lab values were reported. Thus, features that are important for outcome prediction at the time of admission will differ from those that do a better job of modeling variations in disease severity over time. In addition, we stratified our negative outcomes into mortality and morbidity, and separated morbidity further to compare those requiring ICU admission versus ventilation. Eosinophil percentage was statistically different between all three clinical outcomes, while temperature and pulse were only different for morbidity and platelet counts were only different for mortality (Multimedia Appendix 2). It is hypothesized that patients exhibiting symptoms of fever and increased pulse rate, likely a consequence of decreased SpO_2_ (*r*=–0.21 and –0.12, respectively), will likely be prioritized for ICU care and/or ventilation. Although SpO_2_ and respiration rate were consistently selected as predictive features across outcomes and modeling methods, age groups were informative predictors of mortality risk only. As expected, the mortality model performed better than the morbidity models. These results suggest that disease severity and mortality risks may require unique modeling with different predictor subsets and weighting factors. It is also consistent with the observation that senior patients were the most vulnerable population, while the mortality rate among the youth was relatively low [11].

In addition, although current evidence suggests that adults with type 2 diabetes mellitus are at increased risk for COVID-19 complications, our XGBoost model did not find a past diagnosis important for predicting morbidity or mortality. Only after incorporating lab features did we identify a positive correlation between exact glucose values and poorer outcomes. Together, this observation suggests that the elevated blood sugar levels observed may be the result of physiological stress triggered by the disease. Indeed, prior work has shown that even when controlled for pre-existing diabetes, hyperglycemia was commonly observed in acutely ill hospitalized patients and linked to poorer outcomes [12,13].

Other lab features also identified routine chemistry data points that shed light on disease pathology. Values of LDH were elevated for all three clinical outcomes, a finding consistent with widespread tissue damage that has been shown in numerous studies to be a predictor of morbidity and mortality in a wide variety of diseases beyond COVID-19 [14-18]. Mortality was also predicted for by BUN. To investigate further the possibility of any relationship to acute kidney injury, we retrained our models with a BUN to creatinine ratio as an additional feature. While correlated with mortality (*r*=0.17), the feature was not selected for by LASSO, and was only of importance when BUN was removed from the training data set. Indeed, recent literature has revealed that BUN is emerging as an independent predictor of mortality in a variety of diseases, including heart failure [19], aortic dissection [20], and acute pancreatitis [21]. It has also been proposed that BUN is an important indicator for metabolic diseases and general nutritional status of patients, explaining its relative importance in the prediction for mortality. The relationship here is unclear and warrants further investigation.

Interestingly, calcium level upon admission was a more important predictor of morbidity in our models than procalcitonin was. As a peptide precursor of calcitonin, a hormone involved in calcium homeostasis, procalcitonin is also an acute phase reactant that has been used historically, albeit controversially, to help diagnose bacterial pneumonia [22-24]. Although many studies [25-27] have described a positive relationship between procalcitonin levels and mortality and morbidity in patients with COVID-19, few have commented on the importance of calcium as a prognostic value, as we have found in our study. Calcium was negatively correlated with all three measured clinical outcomes, which is consistent with other studies linking hypocalcemia with increased morbidity and mortality in patients with COVID-19 [28-30]. Theoretically, hypocalcemia could be a result of increased procalcitonin, since procalcitonin is the precursor of calcitonin whose function is to reduce serum calcium. Interestingly, it has been reported that in a systemic inflammatory response, serum calcitonin does not increase concordantly in response to increased procalcitonin. This situation could indicate that calcium is a predictive factor through an entirely different mechanism than the more well-established procalcitonin. One theory is that alteration of calcium homeostasis is perhaps used as a strategy by the SARS-CoV-2 virus for survival and replication, since calcium is essential for virus structure formation, entry, gene expression, virion maturation, and release. Another possibility is that patients who present with hypocalcemia have pre-existing parathyroid hormone (PTH) and vitamin D imbalances that are exacerbated by SARS-CoV-2 infection. Our study could not evaluate the importance of PTH or vitamin D due to infrequent lab orders (0.21% and 0.08% completeness, respectively).

While the inclusion of lab features resulted in only modest improvement for ventilation and ICU admission prediction, lab values did result in larger increases in performance metrics for mortality prediction. However, time series modeling failed to improve prediction performance with more clinical features. This observation is likely due to the fact that laboratory results were sampled much less frequently than vital sign readings. Moreover, treating *static* demographic information as repeating time series measurements may be suboptimal for recurrent models. As discussed above, laboratory measurements may help in modeling mortality risk of patients, and future work will focus on efficiently incorporating these static features for dynamic predictions [31,32].

A key limitation of our data set revolves around balancing inclusion criteria to maximize the number of encounters available for model training, while also limiting the amount of missing data. For example, patients who test positive for COVID-19 and present with less severe symptoms in outpatient or telehealth settings may not have a complete set of vital signs or any lab values available. Similarly, past medical histories are dependent on an accurate recollection on the patient’s part, either through a past hospital encounter or at the time of admission. In addition, it is possible that the comorbidities designation in our data set may have false negatives. Because patient histories are often self-reported, it is possible that admitted patients with no prior encounters with the hospital, or either the physical or cognitive inability to verbalize such history at the time of triage, would not have such indication available in the electronic health record. However, this reflects real-world medical situations, in which diagnoses must be made based on unverifiable patient data or delayed lab results. Finally, as data were retrospectively gathered from Epic during the early stages of the pandemic, when diagnostic and treatment protocols were still being developed, a concerted effort to gather novel biomarker tests that have later been shown to be linked with disease severity is not expected. As time draws on and new variants emerge, we also expect that repeated studies will be needed to survey changes to risk factors.

## Appendix

Multimedia Appendix 1 Flow diagram to illustrate the number of patients at each filtering step and descriptions of the criteria that needed to be fulfilled.

Multimedia Appendix 2 Demographics, vital signs, lab results, and clinical outcomes of the retrospective cohort.

Multimedia Appendix 3 Effect of early ventilation treatment on vital signs. A. Distribution of averaged respiration rates for all values preceding 24 hours (top) and for all values that precede initiation of ventilation treatment (bottom). B. Distribution of averaged SpO<sub>2</sub> (peripheral oxygen saturation) for all values preceding 24 hours (top) and for all values that precede initiation of ventilation treatment (bottom).

Multimedia Appendix 4 XGBoost (eXtreme Gradient Boosting) parameter settings for each trained model.

Multimedia Appendix 5 Description of features utilized for time series modeling.

## Abbreviations

AUC: area under the curve
BUN: blood urea nitrogen
CRP: C-reactive protein
GRU: gated recurrent unit
HIPAA: Health Insurance Portability and Accountability Act
ICU: intensive care unit
IRB: Institutional Review Board
LASSO: least absolute shrinkage and selection operator
LDH: lactate dehydrogenase
LSTM: long short-term memory
MCIT: Medical Center Information Technology
MLP: multilayer perceptron
NYU: New York University
PCR: polymerase chain reaction
PTH: parathyroid hormone
RNN: recurrent neural network
SpO_2_: peripheral oxygen saturation
XGBoost: eXtreme Gradient Boosting
