# Supplementary figures and images for: Predictive Modeling of Morbidity and Mortality in Patients Hospitalized With COVID-19 and its Clinical Implications: Algorithm Development and Interpretation
Source: J Med Internet Res. 2021 Jul 9;23(7):e29514. doi: 10.2196/29514 (PMC8274681; doi:10.2196/29514)

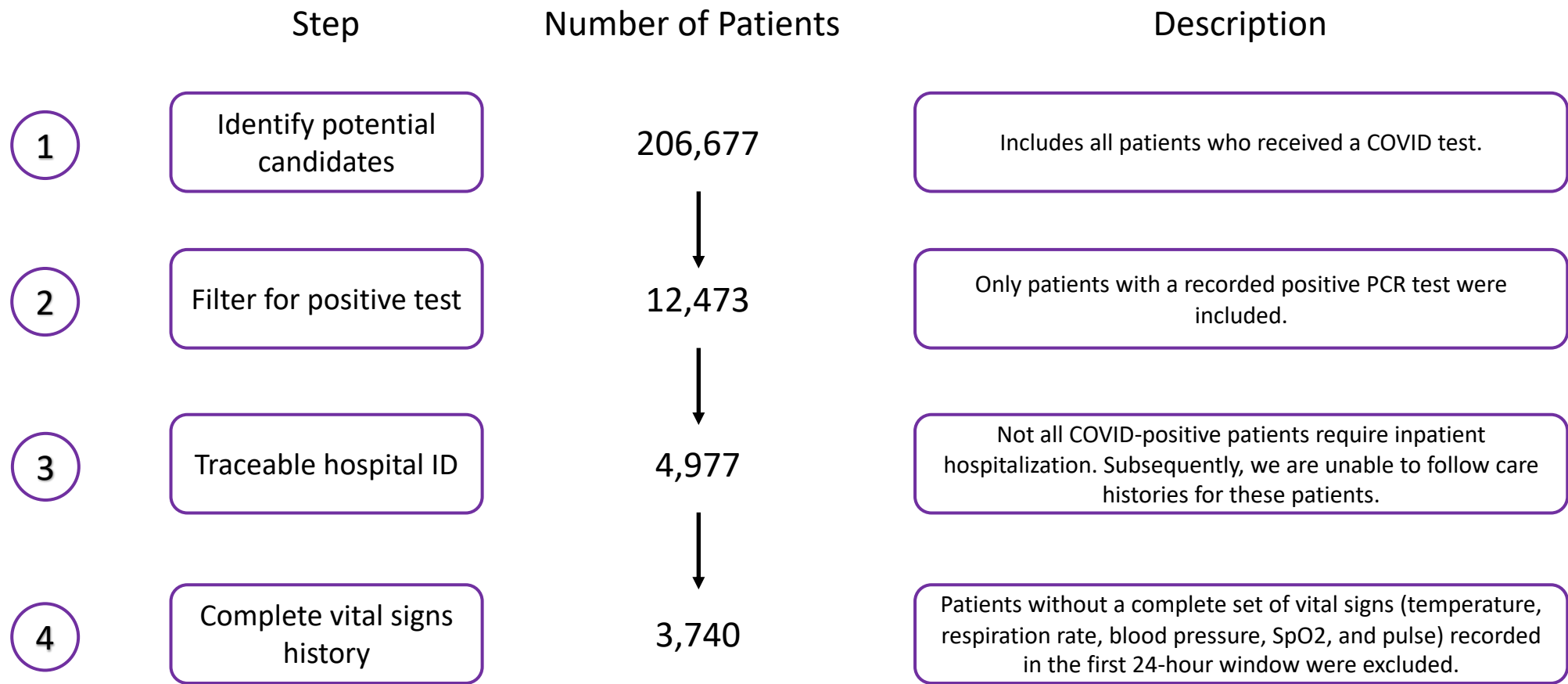

Supplement: Multimedia Appendix 1 [file jmir_v23i7e29514_app1.pdf]

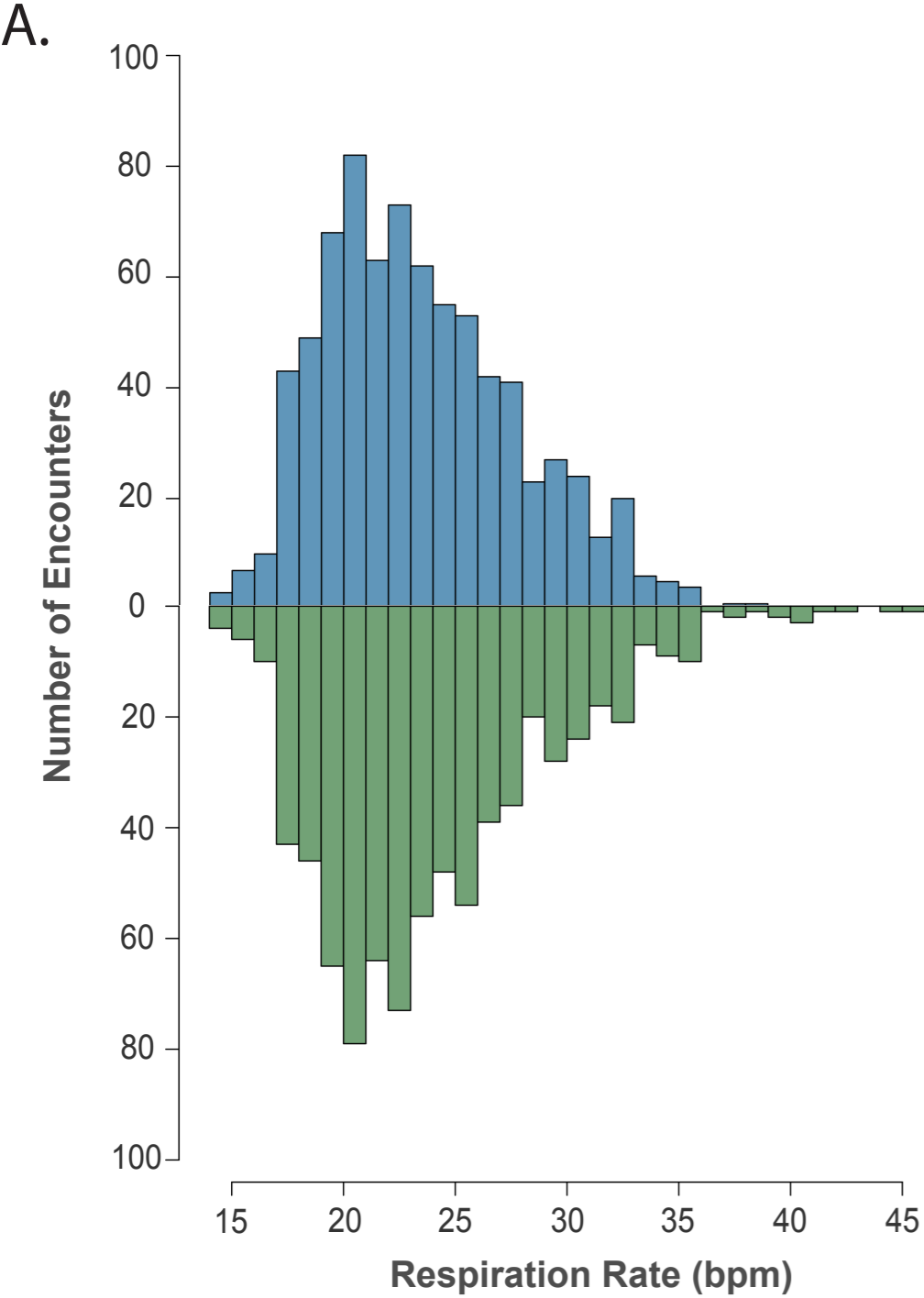

| Respiration Rate (bpm) | Mean (bpm) | SD (bpm) |
|------------------------|------------|----------|
| All                    | 23.63      | 4.35     |
| Filtered Subset        | 24.12      | 5.08     |

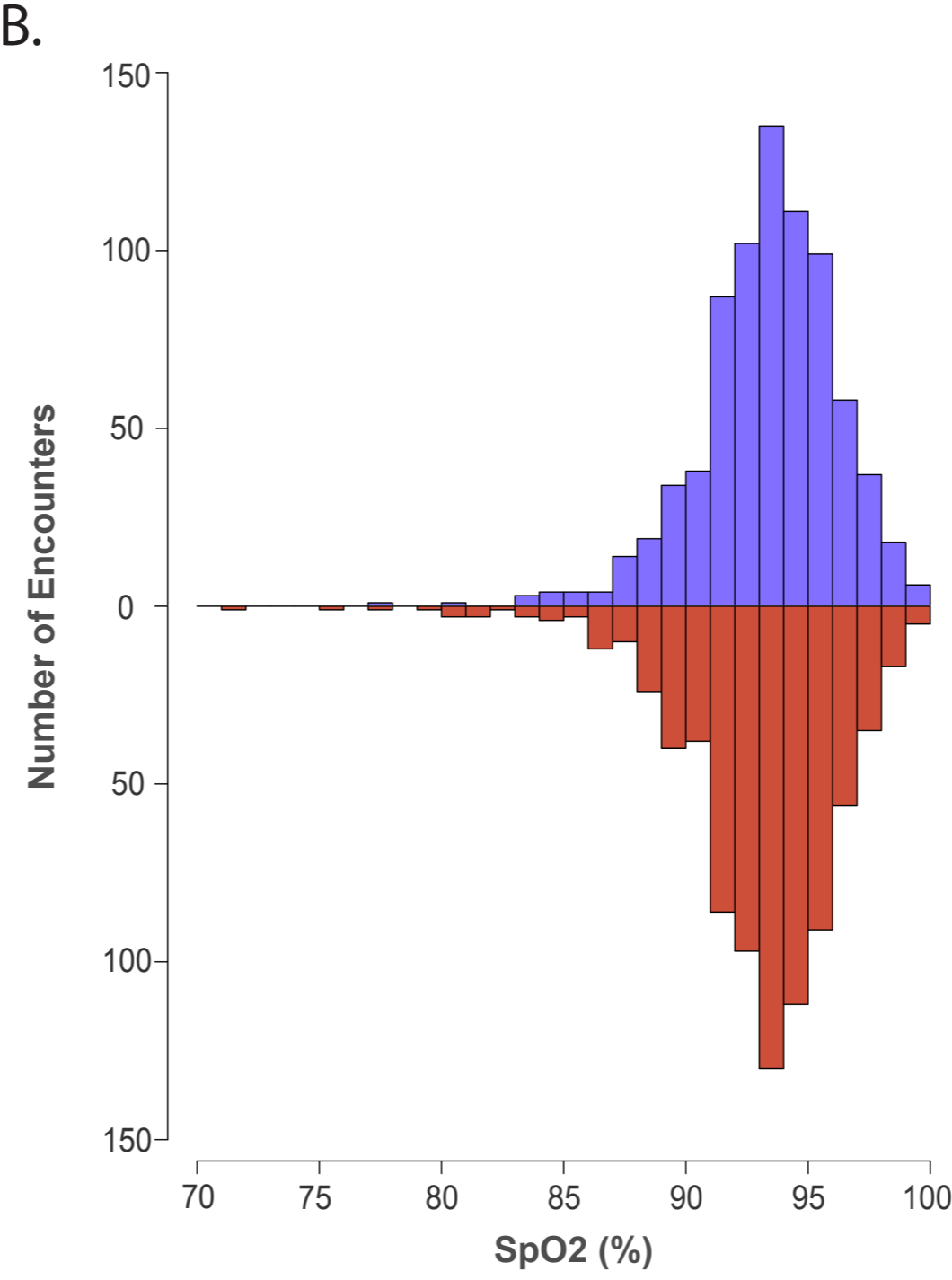

| SpO2 (%)        | Mean (%) | SD (%) |
|-----------------|----------|--------|
| All             | 93.47    | 2.78   |
| Filtered Subset | 93.16    | 3.23   |

Supplement: Multimedia Appendix 3 [file jmir_v23i7e29514_app3.pdf]
